# The Role of Leisure Satisfaction in Serious Leisure and Subjective Well-Being: Evidence From Chinese Marathon Runners

**DOI:** 10.3389/fpsyg.2020.581908

**Published:** 2020-11-10

**Authors:** Hai Bo Tian, Ya Jun Qiu, Ye Qiang Lin, Wen Ting Zhou, Chu Yao Fan

**Affiliations:** ^1^School of Teacher Education, Shaoxing University, Shaoxing, China; ^2^Department of Physical Education, College of Education, Zhejiang University, Hangzhou, China; ^3^Department of Experience Industry Management, California Polytechnic State University, San Luis Obispo, CA, United States

**Keywords:** serious leisure, subjective well-being, leisure satisfaction, mediation effect, marathon runners

## Abstract

The topics of serious leisure and subjective well-being have been discussed extensively in previous research. It is generally acknowledged that people prefer to experience deeper satisfaction and happiness through serious participation in leisure-time physical activities. However, it is essential to examine the relationship between serious leisure and subjective well-being in an urban setting as well as the mediating effect of leisure satisfaction. Data were collected from 447 recreational runners at the 2018 Wuxi International Marathon event in China. The study results showed that serious leisure was positively associated with leisure satisfaction and subjective well-being, that leisure satisfaction was positively associated with subjective well-being, and that leisure satisfaction completely mediated the relationship between serious leisure and subjective well-being. Running group membership significantly affected the path from serious leisure to leisure satisfaction, while other demographic variables (e.g., gender and education) did not moderate any paths. These results help explain the intricate relationship between serious leisure and subjective well-being and offer theoretical and managerial implications for serious leisure.

## Introduction

Due to rapid economic development and improvements in living conditions, leisure has become an essential part of life for urban residents in China. People are no longer satisfied with experiencing short-term, monotonous rewards; instead, they prefer pursuing the long-lasting and multidimensional benefits achieved as they progress in leisure participation (Zhou et al., 2017). Previous literatures documented the benefits of leisure-time physical activity (LTPA) among working adults, clinical populations, deprived communities, children, and older people (Guicciardi et al., 2019; Paggi et al., 2016; Wiese et al., 2018; Won et al., 2020). Under such circumstances, LTPA has rapidly become one of the top choices among many different groups of people pursuing a healthy life. In recent years, marathon running has been considered a prevalent LTPA that caters to the various needs (e.g., healthy lifestyle) of urban residents and has developed into an unprecedented trend in China (Xing et al., 2017). Statistical data indicate that in 2017, approximately 1,100 marathon events involving nearly 5 million participants were held in China, representing an increase of 2.2 million participants compared with the numbers in 2016 (Xiong, 2017).

The concept of serious leisure (SL) was first proposed by Stebbins (1982) based on extensive ethnographic research. This framework advocates that leisure can be understood in a more substantial way than simply ‘free choice’ or ‘free time’ because both perspectives have very limited value (Stebbins, 2007). Marathon running is a challenging and meaningful leisure sport activity that offers an individual a series of durable benefits based on investing a large amount of time and money, improving running knowledge and technology, and overcoming various challenges (Masters et al., 1993; Shipway and Jones, 2007). Through qualitative or quantitative methods, studies have extensively confirmed that marathon running is pursued as a popular SL activity (Shipway and Jones, 2007; Ronkainen N.J. et al., 2017). Although some previous studies have explored the “marathon boom” phenomenon in China (Qin, 2017; Xing et al., 2017) and the development of Chinese marathon events (Wang, 2017; Wu, 2017), few studies have explored the association among LTPA, leisure satisfaction, and subjective well-being using marathon runners as an example, especially from a serious leisure perspective.

The existing literature provides sufficient evidence supporting the role of SL on leisure satisfaction (LS) and subjective well-being (SWB). Stebbins (2007) reported that most SL participants gained a variety of deeper levels of satisfaction (e.g., self-enrichment, self-gratification, or self-actualization) and experienced various positive emotions (e.g., thrill and psychological flow). Previous studies have explored the association between SL and LS and confirmed that significant differences in LS exist between SL and casual leisure (CL) (Cheng, 2010; Yu, 2013; Liu and Yu, 2015). For example, serious participants usually exhibited a higher level of LS, while casual participants exhibited a moderate level of LS. In addition, recent studies have verified that SL is positively associated with SWB, and significant predictors include career and personal effort (Kim et al., 2015) and some durable benefits (Lee and Hwang, 2018). Simultaneously, existing studies have shown that LS is positively related to SWB (Liu, 2014), which has been reconfirmed in samples of college students and senior citizens (Chen, 2008; Cai, 2014). Thus, more empirical research needs to be conducted before confirming the relationship among SL, LS, and SWB. Therefore, this study aims to examine the relationship among SL, SWB, and LS in the Chinese marathon context. This study can extend the application of the SL framework in a different cultural context, contribute to our understanding of the intricate relationships among SL, LS, and SWB, and provide references and implications relevant to both managers of leisure activities and participants.

## Literature Review

### Serious Leisure

SL has been defined as “The systematic pursuit of an amateur, hobbyist, or volunteer activity that is sufficiently substantial and interesting for the participant to find a career there in the acquisition and expression of its special skills and knowledge” (Stebbins, 1992). Stebbins (1992) further described SL based on the following six qualities: perseverance, career, personal effort, unique ethos, strong identity, and durable benefits. Based on the framework proposed by Stebbins, some studies have developed measurement scales to quantify the construct of SL. Gould et al. (2008) developed a 54-item scale named the Serious Leisure Inventory and Measure (SLIM), which comprised 18 subdimensions representing the six significant qualities. Gould et al. (2011) further simplified the 54-item SLIM to a shorter and better-performing 18-item SLIM and found that the short-version SLIM could minimize potential bias and better enhance our understanding of the concept.

Extensive evidence indicates that endurance sports are connected to the qualities of SL. Lamont et al. (2012) indicated that finishing a triathlon (i.e., swimming, cycling, and running) requires good physical fitness and equipment preparation, which are related to participants’ perseverance and personal effort. Lamont and Jenkins (2013) suggested that common elements of cycling events are long distance, duration (hours or days), and terrain (flat or mountain courses) and that recreational cyclists usually cycle under highly regulated competition conditions. Thus, individuals’ SL qualities, including accumulating related knowledge, skill improvement, and physical challenge, are formed during endurance sports experiences (Valenzano et al., 2020).

Distance running (e.g., marathons), which is considered an endurance sport, fits the definition of SL because it requires a time commitment for training, the acquisition of knowledge and skills, and is usually challenging in nature (Wegner et al., 2015). Previous studies have exerted great effort in relating marathons to SL. For instance, based on a quantitative survey, Qiu et al. (2019) found that marathon runners show higher consistency in the six SL qualities. In qualitative interviews, participants in distance events (e.g., Comrades Marathon or Cyprus International Challenge Event) reported significant qualities, such as benefits, unique ethos, and social identity (Shipway and Jones, 2007; Fairer-Wessels, 2013). Overall, considering the rapid growth of marathon events, the increase in the number of participants, and lack of theoretical support in marathon studies, the SL framework is a good fit for studying marathon experiences in China and examining the relationship between SL and other variables (e.g., leisure satisfaction or well-being).

### Serious Leisure and Subjective Well-Being

SWB is defined as an individual’s overall state of subjective wellness and usually includes two primary components: cognitive and affective (Diener, 1984). Over the past three decades, SWB studies have significantly increased in various fields (McMahon, 2008), including leisure activity (Heo et al., 2018), health (Heintzman and Mannell, 2003), and economics (Odermatt and Stutzer, 2017). Existing studies investigating SWB significantly differ in measurement methods and focus on the cognitive component of life satisfaction (Lee and Hwang, 2018), the affective component reflecting individuals’ experiences (Heo et al., 2010) and domain satisfaction (Liu and Yu, 2015), or both (Joshanloo, 2018). Based on a bottom-up approach, recent scholars have focused on identifying the factors influencing SWB, such as motivation (Monacis et al., 2014; Pi et al., 2014), personality (Sato et al., 2018), demographic variables (Diener et al., 1999), community or societal factors (Deaton, 2008), personal circumstances (Luhmann et al., 2012), and degree of leisure involvement (Liu and Yu, 2015).

Several researchers have recognized SL as a significant predictor of SWB. A quantitative study revealed that demographic variables and the qualities of SL can positively predict SWB, and the significant indicators included level of education, self-enrichment, self-expression, and self-gratification (enjoyment) (Lee and Hwang, 2018). Another recent study further confirmed that the qualities of SL (e.g., career contingencies and personal effort) and personal growth had a certain predictive effect on well-being among Taekwondo participants (Kim et al., 2015; Monacis et al., 2015). Chen (2014) explored the effect of spousal support on the relationship between SL and SWB in 264 older adult volunteers in Taiwan. The results of the study confirmed that SL was positively associated with SWB and that spousal support moderated the relationship between SL and SWB. Thus, the following hypothesis is proposed:

Hypothesis 1: Serious leisure is positively associated with subjective well-being.

### Serious Leisure and Leisure Satisfaction

Studies of LS began in the 1980s and aimed to examine individuals’ positive perceptions of leisure participation and overall satisfaction with leisure experiences. Beard and Ragheb (1980) defined LS as “the positive perceptions or feelings which an individual forms, elicits, or gains as a result of engaging in leisure activities and choices, which individuals develop, derive, and acquire from leisure activities and leisure choices.” The Leisure Satisfaction Scale (LSS) developed by Beard and Ragheb (1980) has been widely recognized in leisure research. The LSS includes six subdimensions: psychological, educational, social, relaxation, physiological, and esthetic. This scale has been widely used to measure LS and has exhibited good reliability and validity (Cheng, 2010; Liu and Yu, 2015).

Existing studies have examined the differences in LS among participants with different degrees of leisure involvement. For example, Liu and Yu (2015) compared the differences in LS among members of Chinese college student art groups. Their results revealed significant differences in LS as well as all six subdimensions between the groups, and the SL participants scored higher than the non-SL participants. Since serious running can increase participants’ satisfaction (Shipway and Jones, 2007; Qiu et al., 2019) in the psychological (e.g., self-actualization), educational (e.g., knowledge accumulation), social (e.g., group attraction), relaxation (e.g., relieving stress), physiological (e.g., physical fitness), and esthetic (e.g., well-designed routine) subdimensions, LS may result from the qualities of SL exhibited while running. Therefore, the role of SL in LS in the marathon context must be examined. Thus, the following hypothesis is proposed:

Hypothesis 2: Serious leisure is positively associated with leisure satisfaction.

### Leisure Satisfaction and Subjective Well-Being

Some scholars have made great effort to explore the relationship between LS and SWB, and their results have shown that LS is significantly positively associated with SWB. For example, Liu (2014) examined the relationship among personality, LS, and SWB in SL participants in college student art groups. Their results showed that LS significantly positively affected SWB after controlling for personality characteristics. In addition, existing studies have confirmed that the LS of participants in leisure activities can not only improve their life satisfaction (Agate et al., 2009; Lapa, 2013) but also promote reductions in negative emotions and increase positive emotional experiences (Mannell and Kleiber, 1997; Kim et al., 2015). Chinese scholars have mostly used college students and senior citizens as survey objects and further confirmed the positive effect of LS on SWB (Chen, 2008; Cai, 2014). Thus, the following hypothesis is proposed:

Hypothesis 3: Leisure satisfaction is positively associated with subjective well-being.

### Relationship Among Serious Leisure, Leisure Satisfaction, and Subjective Well-Being

**FIGURE 1:**
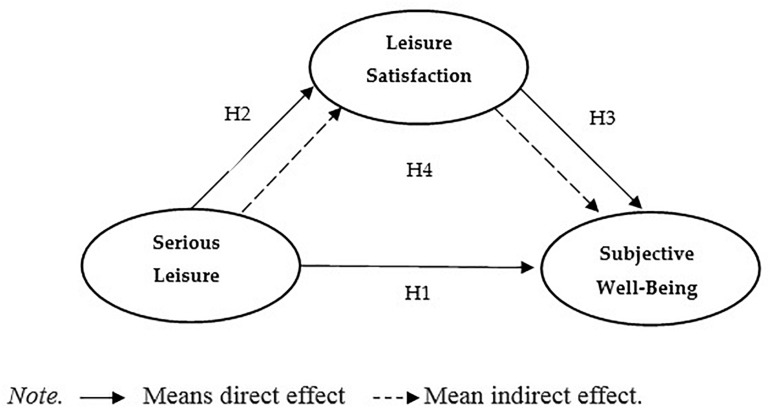
Proposed conceptual model.

Previous research has provided an important background for exploring the relationship among SL, LS, and SWB. Previous research suggests that LS significantly improves as the degree of leisure involvement increases and that SWB improves as LS increases. However, LS may play an important role in the relationship between SL and SWB, and the relationship among the three variables must be re-examined, especially based on the context of a distance running event in China. To address these gaps in the existing literature, this study proposes a mediating model to explore the influence of LS on SL and SWB. Thus, the following hypothesis is proposed (see Figure 1):

Hypothesis 4: Leisure satisfaction can mediate the relationship between serious leisure and subjective well-being.

## Methods

### Date Collection and Analytical Strategies

The data analyzed in this study were collected at the Wuxi International Marathon Event held on March 25, 2018, in Wuxi, a medium-sized city in Southeast China. This event was certified as a gold label event by the China Athletics Association (CAA) in 2015 and has successfully attracted nearly 30,000 participants since 2014. During the questionnaires collection process, the participants were informed about the consent and purpose of the survey, and ethical approval was not required according to the local legislation and institutional requirements. In total, 480 paper questionnaires were randomly (i.e., every 20th person) delivered to half marathon (21.095 km) and full marathon (42.195 km) runners near the finish area. In order to ensure the seriousness of the marathon runners, this study investigated marathon runners who reported running more than three times a week, had run in the past 6 months, and had run more than 5 km once, based on the operational definition suggested in existing studies (Zhou et al., 2017; Qiu et al., 2019). As a result, 447 valid questionnaires were retained for inclusion in the data analysis after removing incomplete and obviously unauthentic questionnaires, yielding a 93.1% overall response rate.

IBM SPSS 22.0 was first used to calculate the descriptive statistics (e.g., frequency, percentage, mean, and standard deviation), Cronbach’s alpha (CA), and correlation analysis. Then, confirmatory factor analysis (CFA) was conducted using AMOS 21 to evaluate the reliability and validity of each construct in the hypothesis model. Third, a linear regression was used to examine Hypothesis 1 and Hypothesis 3, and Hayes (2018) Process-Macro V3.3 in IBM SPSS was utilized to examine Hypothesis 2 and Hypothesis 4.

### Measurements

#### Serious Leisure

SL was measured by the Serious Leisure Inventory and Scale (SLIM) proposed by Gould et al. (2011). Previous studies have used the SLIM to measure outdoor leisure sport activities, such as surfing (Barbieri and Sotomayor, 2013) and rock climbing (Lee et al., 2017), confirming the applicability of this scale. The SLIM consists of six dimensions and comprises the following 18 items: perseverance (1 item), personal effort (1 item), unique ethos (1 item), identity (1 item), career (2 items, career progress and career contingencies), and durable benefits (12 items, e.g., self-expression and self-image). For example, a statement about personal effort is expressed as “I try hard to become more competent in running.” The items were scored using a 5-point Likert scale ranging from 1 (strongly disagree) to 5 (strongly agree). The overall consistency reliability coefficient of the SLIM in this study is 0.95 (see Table 1), similar to the value of 0.97 in the study of Gould et al. (2011).

**TABLE 1:** Mean and statistical analysis of serious leisure, leisure satisfaction, and subjective well-being.

Variables and Dimensions	Mean	SD	FL	CR	AVE
***Serious Leisure (α = *0.95)****	4.11	0.62		0.90	0.61
Perseverance	4.29	0.69	0.74		
Personal effort	4.25	0.78	0.76		
Identity	4.10	0.96	0.73		
Ethos	3.94	0.87	0.69		
Career	4.06	0.72	0.75		
Durable benefits	4.05	0.56	0.97		
***Leisure Satisfaction (α = *0.96)****	4.22	0.53		0.95	0.76
Psychological	4.18	0.61	0.91		
Educational	4.17	0.62	0.91		
Social	4.18	0.65	0.89		
Relaxation	4.33	0.53	0.85		
Physiological	4.32	0.55	0.86		
Esthetic	4.13	0.60	0.81		
***Subjective Well-Being (α = *0.89)****	5.33	1.03		0.91	0.68
SWB 1	5.34	1.10	0.83		
SWB 2	5.62	1.00	0.87		
SWB 3	5.61	1.07	0.88		
SWB 4	5.36	1.22	0.82		
SWB 5	4.71	1.66	0.70		

*SD = standard deviation, FL = factor loading, CR = composite reliability, AVE = average variances extracted.*

#### Leisure Satisfaction

LS was measured by a scale developed by Beard and Ragheb (1980). This scale includes a total of 24 items, including four items related to each of the six dimensions (i.e., psychological, educational, social, relaxation, physiological, and esthetic). The items were scored on a 5-point Likert scale ranging from 1 (strongly disagree) to 5 (strongly agree). The items were slightly revised to fit the characteristics of the marathon. For example, the item “Leisure activity makes me feel confident” was changed to “Running makes me feel confident.” The scale has been confirmed to have good reliability and validity in different cultural backgrounds (Berg et al., 2001; Lysyk et al., 2002; Chen et al., 2013; Lapa, 2013). The overall reliability was 0.96 in this study (see Table 1).

#### Subjective Well-Being

Based on research conducted by Lee and Hwang (2018), SWB was measured by the Satisfaction with Life Scale, which was developed by Diener et al. (1985). The scale includes five items that inquire about the respondents’ evaluation of their global cognitive judgment of their life satisfaction. The items were measured on a 7-point Likert scale ranging from 1 (completely disagree) to 7 (completely agree). An example item is “So far, I have gotten the important things I want in life.” The Cronbach’s alpha reliability coefficient of the SWB in this study was 0.89, suggesting a high level of internal consistency (see Table 1).

Concerning the applicability of the measures above in the Chinese context, first, the measures in English were translated into simplified Chinese by one researcher. Then, another researcher translated the measures back into English. The original meaning of the measures was retained based on a comparison of the two English versions. Second, the final measures were perfected before the formal investigation based on suggestions by three professors who mainly focus on leisure sport studies.

#### Covariate Variables

Previous studies have confirmed that SL, LS, and SWB are associated with related sociodemographic variables (Diener et al., 1999; Liu and Yu, 2015; Qiu et al., 2019). Concerning the potential influence of these variables on the study hypotheses, gender, age, marriage, education, income, and running group were considered covariate variables. Gender was coded with the following 2 categories: 1 = male or 2 = female. Marriage was coded with the following 3 categories: 1 = unmarried, 2 = married, or 3 = divorced or living alone. Age was coded with the following 5 categories: 1 = 19 years and under, 2 = 20–29 years, 3 = 30–44 years, 4 = 45 years-retirement age, or 5 = retirement age. Education was coded with the following 3 categories: 1 = High school or less, 2 = college or university, or 3 = Postgraduate. Income was coded with the following 4 categories: 1 = US$ 3,000 or less, 2 = US$ 3,001-$7,500, 3 = US$ 7,501-$18,000, or 4 = US$ 18,001 or more. Running group was coded with the following 2 categories: 1 = joined or 2 = did not join.

## Results

### Descriptive Statistics of the Variables

The respondents included 131 female runners (29.3% of the sample). Most marathon runners were married (61.3%) and ranged in age from 20 to 44 years (76.9%). Most respondents held a college or postgraduate degree (84.4%), and most respondents had an annual income greater than US $7,500 (72.9%), which is higher than the average income level in the region (i.e., US $6,742). Moreover, most runners were members of a running group (57.9%) (see Table 2).

**TABLE 2:** Demographic descriptive statistics of the marathon runners (*n* = 447).

Demographic Indicators	*n*	*(%)*
**Gender**		
Male	316	70.7
Female	131	29.3
**Marriage**		
Unmarried	167	37.4
Married	274	61.3
Divorced or living alone	6	1.3
**Age**		
19 years or younger	9	2.0
20–29 years	127	28.4
30–44 years	217	48.5
45 years-retirement age	71	15.9
Retirement age or older	23	5.1
**Education**		
High school or less	70	15.7
College or university	261	58.4
Postgraduate	116	26.0
**Income (year)**		
US $3,000 or less	63	14.1
US $3,001–US $7,500	58	13.0
US $7,501–US $18,000	153	34.2
US $18,001 or above	173	38.7
**Running group**		
Joined	259	57.9
Not joined	188	42.1

The marathon runners in this study exhibited relatively high scores on the SL qualities, ranging from 3.94 to 4.29. Perseverance (*M* = 4.29*; SD* = 0.69) and personal effort (*M* = 4.25*; SD* = 0.78) were the qualities on which the marathon runners agreed the most, and the average score of the SL was M = 4.11, SD = 0.62. Similarly, the marathon runners exhibited high scores on LS, ranging from 4.13 to 4.33. The relaxation (*M* = 4.33*; SD* = 0.53) and physiological (*M* = 4.32*; SD* = 0.55) dimensions were the two dimensions on which the marathon runners agreed the most, and the average score of LS was M = 4.22, SD = 0.53. SWLS 2 (*M* = 5.62*; SD* = 1.00) and SWLS 3 (*M* = 5.61*; SD* = 1.07) were scored relatively high, and the average score of SWB was M = 5.33, SD = 1.03 (see Table 2).

The results of confirmatory factor analysis (CFA) verified that all observed variables could be effectively used to evaluate the latent construct in the hypothesized model. Previous research suggests that indicators with factor loadings higher than 0.70 are acceptable (Hair et al., 2011), with a recommended acceptable standard of an AVE value higher than 0.50 and a CR value higher than 0.70 (Fornell and Larcker, 1981). For serious leisure, the factor loadings were between 0.69 and 0.97; the composite reliability (CR) value was 0.90, and the average variances extracted (AVE) was 0.61 (Table 1). The factor loadings of all the sub-dimensions of leisure satisfaction were between 0.81 and 0.91; the CR value was 0.95, and the AVE value was 0.76 (Table 1). For subjective well-being, the factor loadings were between 0.70 and 0.83, the CR value was 0.91, and the AVE value was 0.68 (Table 1). Therefore, the constructs in the hypothesized model exhibited acceptable reliability and validity.

Table 3 shows the correlations among the covariate variables, SL, LS, and SWB. SL was positively correlated with LS (*r* = 0.79, *p* < 0.01) and SWB (*r* = 0.46, *p* < 0.01); LS was positively correlated with SWB (*r* = 0.53, *p* < 0.01); SL, LS, and SWB were correlated with most of the demographic variables, except for the correlations between gender and SL (*r* = −0.09, *p* > 0.05), LS (*r* = −0.05, *p* > 0.05), and SWB (*r* = −0.02, *p* > 0.05), between marriage and LS (*r* = 0.09, *p* > 0.05) and between income and LS (*r* = 0.08, *p* > 0.05) and SWB (*r* = 0.09, *p* > 0.05).

**TABLE 3:** Correlations among demographic variables, serious leisure, leisure satisfaction, and subjective well-being.

Variables	Gender	Age	Education	Marriage	Income	RG	SL	SWB
Age	−0.11*							
Education	0.019	−0.31**						
Marriage	−0.11*	0.60**	−0.21**					
Income	−0.23**	0.21	0.13**	0.29**				
RG	0.11*	−0.23**	0.15**	−0.12*	−0.14**			
SL	−0.09	0.12*	−0.16**	0.10*	0.10*	−0.31**		
SWB	−0.02	0.23**	−0.17**	0.22**	0.09	−0.12**	0.46**	
LS	−0.05	0.12*	−0.17**	0.09	0.08	−0.18**	0.79**	0.53**

RG = running group; SL = serious leisure; LS = leisure satisfaction; SWB = subjective well-being; *p < 0.05; **p < 0.01; ***p < 0.001.

### Hypothesis Testing

Step 1 and Step 3 in Table 4 present the coefficients and significant regression results using SL and LS as independent variables, SWB as dependent variables, and the sociodemographic information as covariate variables. The results indicate that SL (β = 0.445; *P* < 0.001) and age (β = 0.125; *P* < 0.05) were positively associated with SWB; LS (β = 0.506; *P* < 0.001), age (β = 0.107; *P* < 0.05), and marriage (β = 0.104; *P* < 0.05) were positively associated with SWB. Thus, hypotheses 1 and 3 were supported in this study.

**TABLE 4:** Description of the regression and mediation analyses used to test the research hypotheses.

Variables	Steps and Variables (standardized β *and significance*)			
	
	Step 1 (SWB)	Step 2 (LS)	Step 3 (SWB)	Step 4 (SWB)
SL	0.445***	0.815***	–	0.089
LS	–	–	0.506***	0.438***
Gender	0.044	0.016	0.035	0.038
Age	0.125*	0.033	0.107*	0.110*
Education	−0.052	−0.048	−0.032	−0.031
Marriage	0.097	−0.012	0.104*	0.102**
Income	0.011	0.018	0.004	0.003
RG	0.059	0.085**	0.007	0.022
F	21.528***	115.149***	29.584***	26.138***
R^2^	0.256	0.647	0.321	0.323

SL = serious leisure; LS = leisure satisfaction; SWB = subjective well-being; RG = running group. *p < 0.05; **p < 0.01; ***p < 0.001. Step 1 and Step 3 are the results of the regression analyses; Step 2 and Step 4 are the results of Process macro V 3.3, bootstrap proportion is 95%.

Step 2 and Step 4 in Table 4 display the coefficients and significant results (e.g., using model 4 of Process Macro) of SWB using LS as the mediating variable. The results show that SL (β = 0.815; *P* < 0.001) and RG (β = 0.085; *P* < 0.01) were positively associated with LS. LS (β = 0.438; *P* < 0.001), age (β = 0.110; *P* < 0.05), and marriage (β = 0.102; *P* < 0.01) were positively associated with SWB. However, SL (β = 0.089; *P* > 0.05) was not associated with SWB. Concerning the results of step 1, step 4 indicates that LS had a complete mediating effect between SL and SWB. The value of the mediating effect was 0.357 (i.e., 0.815 ^∗^ 0.438). Thus, hypothesis 2 and hypothesis 4 were supported.

This study further examined the effects of demographic variables (i.e., running group, gender, education level, and annual income) on the model path through a series of z-tests. Duncan (1955) argued that if the absolute value of the z-value is greater than 1.96 in a two-tailed test, then this path differs significantly among different groups in a model (p < 0.05). The results of this study showed that the absolute value of z was greater than 1.96 (z = −3.002, p < 0.001) when comparing the effect of the running group on the path from SL to LS. Therefore, it can be inferred that SL has a more significant impact on LS in the model of marathon runners who were part of a running group. However, gender (z = −1.199∼0.784), education (z = −0.809∼0.996), and income (z = −0.997∼0.487) had no significant effect on the three paths (see Table 5).

**TABLE 5:** Comparison of the effects of running group, gender, educational level and annual income on the paths in the model.

Paths	Running Group	Gender	Education	Income
SL→LS	a1-a2 = −3.002***	a1-a2 = −1.199	a1-a2 = −0.585 a1-a3 = −0.809 a2-a3 = −0.382	a1-a2 = −0.586 a1-a3 = −0.810 a1-a4 = −0.566 a2-a3 = −0.382 a2-a4 = 0.084 a3-a4 = 0.487
LS→SWB	b1-b2 = 0.356	b1-b2 = 0.784	b1-b2 = −0.289 b1-b3 = −0.729 b2-b3 = −0.627	b1-b2 = −0.290 b1-b3 = −0.730 b1-b4 = −0.342 b2-b3 = −0.628 b2-b4 = −0.061 b3-b4 = −0.627
SL→SWB	c1-c2 = −0.253	c1-c2 = −0.529	c1-c2 = 0.519 c1-c3 = 0.996 c2-c3 = 0.729	c1-c2 = −0.520 c1-c3 = −0.997 c1-c4 = −0.730 c2-c3 = −0.382 c2-c4 = 0.165 c3-c4 = −0.659

a, b, and c represent the three paths in the model: Running group (1 = joined running group, 2 = did not join running group); Gender (1 = male, 2 = female); Education (1 = High school or less, 2 = College or university, 3 = Postgraduate); Income (1 = $3,000, 2 = $3,001-$7,500, 3 = $7,501-$18,000, 4 = $18,001 and above); ***p < 0.001.

## Discussion

This study mainly examined the interrelationships among SL, LS, and SWB through a regression analysis and Process macro V3.3 for IBM SPSS. First, involvement in a distance running event had a positive influence on the individuals’ life satisfaction (Sato et al., 2018). The results of this study showed that SL was positively associated with SWB among marathon runners, which is consistent with the viewpoints of recent studies (Kim et al., 2015; Lee and Hwang, 2018). Previous studies have also confirmed that serious leisure qualities (e.g., career, personal effort, and durable benefits) were significant predictors of SWB. In contrast to a recent study (Lee and Hwang, 2018), the education level of the participants was not significantly associated with SWB. Weiss (2001) also suggested that distance running experience offers participants various rewards and a meaningful life regardless of differences in gender, ability, or education.

Consistent with the hypotheses in this study, SL was positively associated with LS in marathon running. This result filled the gap between SL and LS and expanded the quantitative findings reported by Liu and Yu (2015), who mainly discussed the difference in LS between participants in SL or non-SL groups. These results also provided sufficient evidence for a theoretical description and qualitative conclusion regarding the existing literature. Stebbins (2007) disclosed that various deep fulfillments were framed in a serious leisure pursuit, which were accompanied by continuous perseverance or significant personal effort. Recent narrative studies have shown that individuals obtain a great degree of satisfaction through serious distance running, such as social relationships or a shifting meaning in running (Ronkainen N. et al., 2017). In addition, consistent with the qualitative results reported by Robinson et al. (2014), belonging to a running group was a positive predictor of LS in this study. As an important social world (Unruh, 1979), running groups can provide more opportunities or information, enhancing the serious running experience and leisure satisfaction.

Another finding from this study is that the LS of marathon runners positively influenced their SWB. The current study enriches the previous literature (Chen, 2008; Cai, 2014; Liu, 2014) by examining the relationship between LS and SWB in marathon participants. When individuals seriously committed and specialized in their leisure pursuit, activity participation became a central lifestyle (Scott and Shafer, 2001). Evidence suggests that individuals express a higher level of happiness when they are satisfied with a running route, event atmosphere, or physical improvement (Shipway and Jones, 2008). Furthermore, in accordance with a previous review (Diener et al., 1999), the findings support age and marriage as important predictors of SWB. The characteristics of distance running may attract a specific group (i.e., older-aged or married individuals), and these individuals experienced a higher degree of SWB while running.

The results of Process macro V3.3 indicated that LS had a mediation effect on the relationship between SL and SWB. This finding indicates that SL is more likely to influence individuals’ LS with running, thereby increasing their SWB. This finding extends the existing literature (Kim et al., 2015; Lee and Hwang, 2018) by introducing LS as a mediation variable and provides a deeper understanding of the relationship between SL and SWB. These findings also extend Liu and Yu (2015) work by examining the association among SL, LS, and SWB. These results further suggest that individuals should adopt different negotiation strategies to achieve their leisure needs, which can not only strengthen their serious running participation behavior but also increase their well-being. Therefore, how to improve participants’ leisure satisfaction in the process of SL participation is an important research theme.

In addition, this study examined the impact of demographic variables (e.g., running group, gender, education, or income) on the path of the theoretical model. The study results indicated that marathon runners differed significantly on the path from SL to LS in the model based on their membership in a running group. As Unruh (1979) described, leisure communities usually have specific norms, values, and beliefs. Such communities can promote an individual behavioral process of acquisition through indirect experience and in turn improve LS at multiple psychological, educational, and societal levels. However, other demographic variables (e.g., gender, education, and annual income) had no significant impact on any of the pathways. These results confirmed the viewpoint of Weiss (2001), who posited that few other social community offer such a level of identity and praise as running in modern society, regardless of gender, age, social status, or education level.

## Conclusion and Implications

This study further extends the application of the SL framework to the leisure sports context among Chinese distance running event participants. A recent study confirmed that SL qualities were positively associated with marathon running participation behaviors, such as the number of running years, running frequency per week, and longest marathon event (Qiu et al., 2019). Based on evidence provided by Liu and Yu (2015), the results of this study contribute to the existing literature by confirming the relationship among SL, LS, and SWB, thus enhancing our understanding of the mechanism underlying the relationship between SL and SWB.

Four research hypotheses were proposed in this study. First, consistent with previous studies (Chen, 2014; Kim et al., 2015; Lee and Hwang, 2018), the findings indicate that SL is positively associated with SWB and extend this relationship to participation in a distance running event (e.g., marathon). In addition, this study confirmed that SL was positively associated with LS. This finding not only supports the results reported by Stebbins (2007) but also extends the results reported by Liu and Yu (2015). Third, consistent with existing studies (Chen, 2008; Cai, 2014; Liu, 2014), LS was positively associated with SWB. This study enriches existing studies by examining the hypotheses among participants with a wider age range (e.g., from 19 years to retirement age and older). Finally, this study found that LS completely mediated the relationship between SL and SWB, extending the results of an existing study (Pi et al., 2014; Liu and Yu, 2015) and contributing to our understanding of the mechanism by which participants gain SWB through serious leisure participation.

This study has several limitations. First, this study examined the relationship among SL, LS, and SWB based on marathon running in China. Future research should be applied to other leisure sports activities and should further examine the hypothetical model. Second, this study verified the mediation effect of LS on the relationship between SL and SWB. Other variables (e.g., flow experience and recreation specialization) should be explored via the mediation model in subsequent studies. Third, this study examined the mediation effect of LS using the total SL, LS, and SWB scores; future studies should explore the relationship among the subdimensions of the three variables and consider the mediation effect of other variables (e.g., recreation specialization and leisure involvement) in the model in this study.

These results have theoretical and practical implications for the field of leisure activities. First, many variables may mediate or moderate the influence of SL on SWB. Scholars should focus on exploring these variables through both quantitative and qualitative methods to discover the potential framework that affects participants’ well-being. Second, organizers of marathon events should cater to the various needs of participants (e.g., esthetic, social, and educational) and provide high-quality service in various aspects, such as security, energy supply, and route design. The more LS marathon runners acquire, the higher their SWB, and the more likely they are to participate in marathons again in the future. Third, government agencies (e.g., general administrations for sports) should provide more support to people, such as perfecting the running route, strengthening the construction of running groups, and optimizing relevant policies. These changes could promote individuals’ serious participation in running activities and offer a deeper degree of LS and SWB.

## Data Availability Statement

The raw data supporting the conclusions of this article will be made available by the authors, without undue reservation.

## Ethics Statement

Ethical review and approval was not required for the study on human participants in accordance with the local legislation and institutional requirements. Written informed consent from the participants was not required to participate in this study in accordance with the national legislation in the host country and the institutional requirements.

## Author Contributions

HT wrote this manuscript. YQ was responsible for research design and ideas. YL was responsible for research revision. WZ and CF were responsible for collecting questionnaire and analyzing the data. All authors contributed to the article and approved the submitted version.

## Conflict of Interest

The authors declare that the research was conducted in the absence of any commercial or financial relationships that could be construed as a potential conflict of interest.
